# Data on polymorphism of XRCC1 and cervical cancer risk from South India

**DOI:** 10.1016/j.dib.2016.11.052

**Published:** 2016-11-26

**Authors:** Geethakumari Konathala, Ramesh Mandarapu, Sudhakar Godi

**Affiliations:** Department of Human Genetics, Andhra University, Visakhapatnam 530003, Andhra Pradesh, India

**Keywords:** XRCC1, Polymorphism, DNA Repair Genes, Indian population

## Abstract

X-ray repair cross-complementing group 1 (XRCC1) is a major DNA repair gene involved in BER mutation. Polymorphisms in DNA repair genes associated with repair efficiency against DNA damage may predispose an individual׳s cancer susceptibility. Data from cervical cancer patients was collected from South Indian Women. Genotyping of XRCC1 polymorphisms (194C/T, 280G/A and 399G/A) was done by polymerase-chain-reaction with the confronting-two-pair primer (PCR-CTPP) method.

**Specifications Table**

**Table:** 

Subject area	Biology
More specific subject area	Molecular Genetics
Type of data	Tables, graphs, figures
How data was acquired	Survey, Collection of blood samples, Gradient PCR
Data format	Analyzed
Experimental factors	Isolation of genomic DNA from peripheral blood.
Experimental features	Genotyping of three SNPs by polymerase-chain-reaction with the confronting-two-pair primer (PCR-CTPP) method.
Data source location	Mahatma Gandhi cancer Hospital, King George Hospital and Lions Cancer Hospital, Visakhapatnam, Andhra Pradesh, India.
Data accessibility	Data are presented in this article

**Value of the data**

• Only few works on cervical cancer have been carried out from India. Especially, no data is available on DNA repair genes polymorphisms of cervical cancer from north coastal Andhra Pradesh. This data may fill the gap from this region.
• To acquire the knowledge of XRCC1 allelic profiles and genotype distribution in the normal population and patients to observe their association with risk of cervical cancer.
• The Multifactor Dimensionality Reduction (MDR) method was used to assess higher order gene-gene interactions that may confer high or low-risk for cervical cancer development using genotype data on three SNPs.
• This data is useful to determine the genetic diversity from this region.

## 1 Data

Tables 1 and 2 describe the Association, Relative Risk and Odds Ratio of XRCC1 (194C>T), (280G>A), (399G>A) genotypes in disease and control groups. Table 3 summarizes the comparative table of polymorphisms of DNA repair gene XRCC1. Graphs 1 and 2 represent the best double and three-locus bar diagrams. Fig. 1 illustrates the MDR interaction information analysis of the three polymorphisms, represented in the form of a dendrogram. Pairwise linkage disequilibrium (LD) for three SNPs were calculated. The analysis has generated 8 marker combinations from three SNPs in both cases and controls (Table 4; Graph 3).

## 2 Experimental design, materials and methods

### 2.1 Subjects

This research was designed to be a case-control study. The study was ethically approved for collecting blood samples from the human subjects by the local [Andhra University] ethics committee. 125 patients who were diagnosed with cervical cancer at King George Government Hospital, Lions Cancer Hospital and Mahatma Gandhi Cancer Hospital and Research Institute, Visakhapatnam, Andhra Pradesh, India were recruited. Around 150 control samples were collected and analyzed for the molecular parameters.

### 2.2 DNA extraction

Genomic DNA was obtained from 1 ml of EDTA anticoagulated whole blood by the salting-out method with slight modifications [1]. Both cases and controls were genotyped in a randomized, blinded fashion.

### 2.3 Determination of XRCC1 genotype

Genotyping of XRCC1 polymorphisms (194C/T, 280G/A and 399G/A) was made by polymerase-chain-reaction with the confronting-two-pair primer (PCR-CTPP) method [2]. PCRs were carried out in a total volume of 15 μl.

The primers are as follows:

**Table:** 

194C/T	NF: 5′ CCCTTTGGCTTGAGTTTTGT 3′;	MF: 5′ GGGCTCTCTTCTTCAGCT 3′
	NR: 5′ GGGATGTCTTGTTGATCCG 3′;	MR:5′ TGCTGGGTCGCTGGCTGTG 3′
280G/A	NF: 5′ CTCTTCCCAAGAGACCTAAAT 3′;	MF: 5′ TCCAGTGCCAGCTCCAACTCA 3′
	NR: 5′ ACTGGGGCTGTGGCTGGGGTAC 3′;	MR: 5′ TAGGGCCTTATCTCGCAGCTC 3′
399G/A	NF: 5′ ATCCTTCAGGGTGTGGTAGTG 3′;	MF: 5′ GTCGGCGGCTGCCCTCCCG 3′
	NR: 5′ TGGCGTGTGAGGCCTTACCTCT 3′;	MR: 5′ TCCCACCCCTGAGTTTTTGCAC 3′

The primers were designed by using primer 3plus software (http://primer3plus.com/cgi-bin/dev/primer3plus.cgi). PCR amplification was performed in a thermal cycler gradient PCR system (Lark, India). The PCR amplification was performed for 30 cycles (denaturation at 95 °C for 20 s, annealing for 30 s at 56 °C, extension at 72 °C for 20 s and final extension for 5 min at 72 °C. PCR products of 608 bp (194 C/T), 747 bp (280 G/C) and 837 bp (399 G/C) were analyzed by 1.5% agarose gel stained with ethidium bromide.

## Footnotes

Appendix A Supplementary data associated with this article can be found in the online version at doi:10.1016/j.dib.2016.11.052.
