# Supplementary material for: Data on polymorphism of XRCC1 and cervical cancer risk from South India
Source: Data Brief. 2016 Nov 26;10:11–3. doi: 10.1016/j.dib.2016.11.052 (PMC5133639; doi:10.1016/j.dib.2016.11.052)
Supplement: Supplementary file 2 — Supplementary material [file mmc1.docx]

***Conflicts of Interest Statement***

Manuscript title: **Data on Polymorphism of XRCC1 and Cervical cancer risk from South India**

Author names: **Geethakumari Konathala*, Ramesh Mandarapu and Sudhakar Godi**

Affiliations: Department of Human Genetics, Andhra University, Visakhapatnam – 530003,

Andhra Pradesh, India.

This statement is signed by all the authors to indicate agreement that the above information is true and cor­rect **(a photocopy of this form may be used if there are more than 10 authors):**

Author's name (typed) Author's signature Date

Geethakumari Konathala
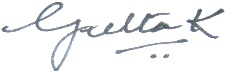
 27.09.2016

Ramesh Mandarapu
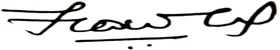
 27.09.2016

Sudhakar Godi
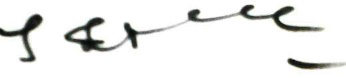
 27.09.2016
