# Supplementary material for: Data on polymorphism of XRCC1 and cervical cancer risk from South India
Source: Data Brief. 2016 Nov 26;10:11–3. doi: 10.1016/j.dib.2016.11.052 (PMC5133639; doi:10.1016/j.dib.2016.11.052)
Supplement: Supplementary file 1 — Supplementary material [file mmc2.zip › Supplementary Figure 1.doc]

**Figure 1: A tree diagram of the interactions among three factors XRCC1 (194, 280 and 399) for Cervical cancer, as analyzed by MDR.**

**280**

**194**

**399**

**Weak Interactions	Strong Interactions**
