# Supplementary material for: Data on polymorphism of XRCC1 and cervical cancer risk from South India
Source: Data Brief. 2016 Nov 26;10:11–3. doi: 10.1016/j.dib.2016.11.052 (PMC5133639; doi:10.1016/j.dib.2016.11.052)
Supplement: Supplementary file 1 — Supplementary material [file mmc2.zip › Supplementary Graph 1.doc]

**Graph 1: An MDR analysis of the two factors (XRCC1_194 and 399) interaction model of Cervical Cancer**

**Bar represents distribution of cases (left) and controls (right) with each multifactor combination**

(a) **Dark shaded cells – High-risk genotype combination**
(b) **Lightly shaded cells – Low-risk genotype combination**
(c) **No shading or white cells – genotypic combinations for which no data was observed.**
