# Supplementary material for: Data on polymorphism of XRCC1 and cervical cancer risk from South India
Source: Data Brief. 2016 Nov 26;10:11–3. doi: 10.1016/j.dib.2016.11.052 (PMC5133639; doi:10.1016/j.dib.2016.11.052)
Supplement: Supplementary file 1 — Supplementary material [file mmc2.zip › Supplementary Graph 3.doc]

**Graph 3: LD plot showing interaction between two gene polymorphisms in**

**Controls	Patients**

1. **XRCC1 194C/T**
2. **XRCC1 280G/A**
3. **XRCC1 399G/A**
