# Supplementary material for: Data on polymorphism of XRCC1 and cervical cancer risk from South India
Source: Data Brief. 2016 Nov 26;10:11–3. doi: 10.1016/j.dib.2016.11.052 (PMC5133639; doi:10.1016/j.dib.2016.11.052)
Supplement: Supplementary file 1 — Supplementary material [file mmc2.zip › Supplementary Table 1.doc]

**Table 1: Analysis of association between XRCC1 (194C>T), (280G>A), (399G>A) polymorphism and risk for cervical carcinoma**

| **Gene** | **Genotype & Alleles** | **Patients (N=125)** | | **Controls (N=150)** | | **Chi-square (χ^2^)** | |
| --- | --- | --- | --- | --- | --- | --- | --- |
|  |  | **Obs** | **Exp** | **Obs** | **Exp** | **Patients** | **Controls** |
| **XRCC1**  **194** | **CC** | 82.00 | 80.00 | 40.00 | 45.40 | **1.2500^NS^**  (0.30>p>0.20) | **3.1442^NS^**  (0.30>p>0.20) |
|  | **CT** | 36.00 | 40.00 | 85.00 | 74.30 |  |  |
|  | **TT** | 07.00 | 05.00 | 25.00 | 30.30 |  |  |
|  | **C**  **T** | 0.8000 ± 0.0225  0.2000 ± 0.0225 | | 0.5500 ± 0.0158  0.4500 ± 0.0158 | | **Inter Group χ^2^**  **χ^2^= 5.067^NS^** (0.10>p>0.05) | |
| **XRCC1**  **280** | **GG** | 71.00 | 73.00 | 75.00 | 74.20 | **χ^2^= 0.9473^NS^**  (0.50>p>0.30) | **χ^2^=0.0975^NS^**  (0.80>p>0.70) |
|  | **GA** | 49.00 | 45.00 | 61.00 | 62.60 |  |  |
|  | **AA** | 05.00 | 07.00 | 14.00 | 13.20 |  |  |
|  | **G**  **A** | 0.7600 ± 0.0264  0.2400 ± 0.0264 | | 0.7000 ± 0.0264  0.3000 ± 0.0264 | | **Inter Group χ^2^**  **χ^2^= 3.444^NS^** (0.20>p>0.10) | |
| **XRCC1**  **399** | **GG** | 65.00 | 57.80 | 55.00 | 52.20 | **χ^2^= 8.7586^**^**  (0.01>p>0.001) | **χ^2^=0.8836^NS^**  (0.50>p>0.30) |
|  | **GA** | 40.00 | 54.40 | 67.00 | 72.60 |  |  |
|  | **AA** | 20.00 | 12.80 | 28.00 | 25.20 |  |  |
|  | **G**  **A** | 0.6800 ± 0.0264  0.3200 ± 0.0264 | | 0.5900 ± 0.0264  0.4100 ± 0.0264 | | **Inter Group χ^2^**  **χ^2^= 6.763*** (0.05>p>0.01) | |

***p<0.05, **p<0.01, ***p<0.001, NS Non-significant**
