# Supplementary material for: Data on polymorphism of XRCC1 and cervical cancer risk from South India
Source: Data Brief. 2016 Nov 26;10:11–3. doi: 10.1016/j.dib.2016.11.052 (PMC5133639; doi:10.1016/j.dib.2016.11.052)
Supplement: Supplementary file 1 — Supplementary material [file mmc2.zip › Supplementary Table 2.doc]

**Table 2: Test of Association, Relative Risk, Odds Ratio and 95% Confidence Interval estimates of XRCC1 (194C>T), (280G>A), (399G>A) genotypes in disease and control groups**

| **Gene** | **Genotype combinations** | | **Disease and Control Groups** | | | |
| --- | --- | --- | --- | --- | --- | --- |
|  |  |  | **RR** | **OR (95%CI)** | **χ^2^ value** | **p- value** |
| **XRCC1 194** | **Homozygote Comparison** | TT *vs* CC | 0.33 | 0.14 (0.05-0.37) | 21.36 | 0.0000******* |
|  | **Heterozygote Comparison** | CT *vs* CC | 0.44 | 0.21 (0.12-0.37) | 34.13 | 0.0000******* |
|  | **Dominant Model** | CT/TT *vs* CC | 0.42 | 0.19 (0.11-0.33) | 41.87 | 0.0000******* |
|  | **Recessive Model** | TT *vs* CT/CC | 0.45 | 0.30 (0.11-0.75) | 8.12 | 0.0043******* |
|  | **Allele contrast** | T *vs* C | 0.52 | 0.31 (0.15-0.60) | 14.25 | 0.0001******* |
| **XRCC1**  **280** | **Homozygote Comparison** | AA *vs* GG | 0.54 | 0.38 (0.11-1.20) | 3.37 | 0.0664 |
|  | **Heterozygote Comparison** | GA *vs* GG | 0.92 | 0.85 (0.50-1.44) | 0.42 | 0.5168 |
|  | **Dominant Model** | GA/AA *vs* GG | 0.86 | 0.76 (0.46-1.26) | 1.27 | 0.2605 |
|  | **Recessive Model** | AA *vs* GG/GA | 0.56 | 0.40 (0.12-1.25) | 3.02 | 0.0825 |
|  | **Allele contrast** | A *vs* G | 0.85 | 0.74 (0.38-1.44) | 0.91 | 0.3392 |
| **XRCC1**  **399** | **Homozygote Comparison** | AA *vs* GG | 0.77 | 0.60 (0.29-1.25) | 2.14 | 0.1432 |
|  | **Heterozygote Comparison** | GA *vs* GG | 0.69 | 0.51 (0.29-0.89) | 6.41 | 0.0113** |
|  | **Dominant Model** | GA/AA vs GG | 0.71 | 0.53 (0.32-0.89) | 6.52 | 0.0106****** |
|  | **Recessive Model** | AA \*vs* GG/GA | 0.90 | 0.83 (0.42-1.63) | 0.34 | 0.5618 |
|  | **Allele contrast** | A *vs* G | 0.82 | 0.68 (0.36-1.26) | 1.75 | 0.1862 |

***p<0.05, **p<0.01, ***p<0.001, NS Non-significant**
