# Supplementary material for: Data on polymorphism of XRCC1 and cervical cancer risk from South India
Source: Data Brief. 2016 Nov 26;10:11–3. doi: 10.1016/j.dib.2016.11.052 (PMC5133639; doi:10.1016/j.dib.2016.11.052)
Supplement: Supplementary file 1 — Supplementary material [file mmc2.zip › Supplementary Table 3.doc]

| **Table 3: A comparative table of XRCC1 (194C>T), (280G>A), (399G>A) polymorphism** | | | | | | | | | | | | | | | | |
| --- | --- | --- | --- | --- | --- | --- | --- | --- | --- | --- | --- | --- | --- | --- | --- | --- |
| **XRCC1_194** | | | | | | | | | | | | | | | | |
| **Sn** | **Author** | **year** | **country** | **Ethnicity** | **Case/**  **cntrl (No.)** | **Ca-Cx Patients** | | | | | **Controls** | | | | | **Pairwise χ^2^ value between Indian & other countries** |
|  |  |  |  |  |  | **Genotypes** | | | **Alleles** | | **Genotypes** | | | **Alleles** | |  |
|  |  |  |  |  |  | **CC** | **CT** | **TT** | **C** | **T** | **CC** | **CT** | **TT** | **C** | **T** |  |
| **1** | **Present Study** | 2015 | India | Asian | 125/150 | 82 | 36 | 7 | 0.80 | 0.20 | 40 | 85 | 25 | 0.55 | 0.45 | **Ref** |
| **2** | Huang | 2007 | China | Asian | 539/800 | - | - | - | 0.65 | 0.35 | - | - | - | - | - | 5.64 |
| **3** | Kidong Kim | 2008 | Korea | Asian | 66/0 | 34 | 23 | 9 | 0.69 | 0.31 | - | - | - | - | - | 3.18^a^ |
| **4** | Farkasova | 2008 | Slovakia | Caucasian | 17/30 | - | - | - | - | - | - | - | - | - | - | - |
| **5** | Wang | 2010 | China | Asian | 123/175 | - | - | - | 0.72 | 0.28 | - | - | - | - | - | 1.75^a^ |
| **6** | Settheetham | 2011 | Thailand | Asian | 111/118 | 53 | 49 | 9 | 0.70 | 0.30 | 65 | 51 | 2 | 0.77 | 0.23 | 2.67^a^ |
| **7** | Barbisan G | 2011 | Argentina | LAmerica | 103/114 | 79 | 20 | 4 | 0.86 | 0.14 | 98 | 12 | 4 | 0.91 | 0.09 | 1.28^a^ |
| **8** | Zhang L | 2012 | China | Asian | 80/177 | 41 | 31 | 8 | 0.71 | 0.29 | 87 | 71 | 19 | 0.69 | 0.31 | 2.19^a^ |
| **9** | Yilmaz | 2012 | Turkey | Turks | 35/10 | 29 | 6 | 0 | 0.91 | 0.09 | 9 | 1 | 0 | 0.95 | 0.05 | 4.88 |
| **10** | Leyla | 2013 | Khazakstan | Caucasia | 217/160 | 163 | 48 | 6 | 0.86 | 0.14 | 105 | 40 | 15 | 0.78 | 0.22 | 1.28^a^ |
| **XRCC1_280** | | | | | | | | | | | | | | | | |
| **1** | **Present Study** | 2015 | India | Asian | 125/150 | 71 | 49 | 5 | 0.76 | 0.24 | 75 | 61 | 14 | 0.70 | 0.30 | **ref** |
| **2** | Huang | 2007 | China | Asian | 539/800 | - | - | - | 0.88 | 0.12 | - | - | - | 0.88 | 0.12 | 4.88 |
| **3** | Wang | 2010 | China | Asian | 123/175 | - | - | - | 0.86 | 0.14 | - | - | - | 0.90 | 0.10 | 3.25^a^ |
| **4** | Zhang L | 2012 | China | Asian | 80/177 | 68 | 11 | 1 | 0.92 | 0.08 | 142 | 34 | 1 | 0.90 | 0.10 | 9.52 |
| **XRCC1_399** | | | | | | | | | | | | | | | | |
| **1** | **Present Study** | 2015 | India | Asian | 125/150 | 65 | 40 | 20 | 0.68 | 0.32 | 55 | 67 | 28 | 0.59 | 0.41 | **Ref** |
| **2** | Wu | 2004 | Taiwan | Asian | 100/196 | 54 | 38 | 8 | 0.73 | 0.27 | 114 | 73 | 9 | 0.77 | 0.23 | 0.60^a^ |
| **3** | Niwa Y | 2005 | Japan | Asian | 131/320 | 69 | 49 | 13 | 0.71 | 0.29 | 185 | 109 | 26 | 0.75 | 0.25 | 0.21^a^ |
| **4** | Huang | 2007 | China | Asian | 539/800 | 289 | 203 | 47 | 0.72 | 0.28 | 528 | 235 | 37 | 0.81 | 0.19 | 0.38^a^ |
| **5** | Hong | 2008 | China | Asian | 72/176 |  |  |  | 0.74 | 0.26 |  |  |  | 0.78 | 0.22 | 0.87^a^ |
| **6** | Farkasova | 2008 | Slovakia | Caucasia | 17/30 | - | - | - | - | - | - | - | - | - | - | - |
| **7** | Xiao-Dong Cheng | 2009 | China | Asian | 70/- | 44 | 24 | 2 | 0.80 | 0.20 | - | - | - | - | - | 3.74 |
| **8** | Jiang W | 2009 | China | Asian | 436/503 | 228 | 184 | 24 | 0.73 | 0.27 | 268 | 194 | 41 | 0.73 | 0.27 | 0.60^a^ |
| **9** | Xiao HY | 2010 | China | Asian | 162/183 | 91 | 56 | 15 | 0.73 | 0.27 | 94 | 68 | 21 | 0.70 | 0.30 | 0.60^a^ |
| **10** | Ma WP | 2011 | China | Asian | 200/200 | 108 | 76 | 16 | 0.73 | 0.27 | 133 | 55 | 12 | 0.80 | 0.20 | 0.60^a^ |
| **11** | Settheetham | 2011 | Thailand | Asian | 111/118 | 66 | 41 | 4 | 0.78 | 0.22 | 69 | 44 | 5 | 0.77 | 0.23 | 2.54^a^ |
| **12** | Barbisan G | 2011 | Argentina | America | 103/114 | 54 | 31 | 18 | 0.67 | 0.33 | 37 | 59 | 18 | 0.58 | 0.42 | 0.02^a^ |
| **13** | Roszak | 2011 | Poland | Caucasia | 189/308 | - | - | - | 0.53 | 0.47 | - | - | - | 0.63 | 0.37 | 4.71 |
| **14** | Zhang L | 2012 | China | Asian | 80/177 | 43 | 31 | 6 | 0.73 | 0.27 | 109 | 58 | 10 | 0.78 | 0.22 | 0.60^a^ |
| **15** | Yilmaz | 2012 | Turkey | Turks | 35/10 | 19 | 13 | 3 | 0.71 | 0.29 | 1 | 6 | 3 | 0.60 | 0.40 | 0.02^a^ |
| **16** | Ghazi Alsbeih | 2013 | Saudi Arabi | Asian | 100/100 | 52 | 34 | 14 | 0.69 | 0.31 | 59 | 40 | 1 | 0.79 | 0.21 | 0.02^a^ |
| **17** | Leyla | 2013 | Khazakstan | Caucasia | 217/160 | 78 | 119 | 20 | 0.63 | 0.37 | 66 | 90 | 4 | 0.69 | 0.31 | 0.55^a^ |

**^a^ Chi-square test statistic value less than 3.841 at 5% significance level, so the other studies are not significantly different from India.**
