# Supplementary material for: Data on polymorphism of XRCC1 and cervical cancer risk from South India
Source: Data Brief. 2016 Nov 26;10:11–3. doi: 10.1016/j.dib.2016.11.052 (PMC5133639; doi:10.1016/j.dib.2016.11.052)
Supplement: Supplementary file 1 — Supplementary material [file mmc2.zip › Supplementary Table 4.doc]

**Table 4: Haplotype frequencies of significant D-loop polymorphisms observed in patients and controls**

| **Haplotypes** | | | **Haplotype Frequency** | | **χ^2^** | ***p* value^a^** | **Odds ratio** | **95% CI** |
| --- | --- | --- | --- | --- | --- | --- | --- | --- |
| **+194** | **+280** | **+399** | **Cases** | **Controls** |  |  |  |  |
| C | G | G | 120 (48.0) | 165 (55.0) | Reference | - | - | - |
| T | G | A | 12 (4.8) | 31 (10.3) | 3.13 | 0.0768 | 1.88 | 0.92-3.81 |
| T | A | A | 10 (4.0) | 84 (29.7) | 31.06 | 0.0000*** | 6.11 | 3.04-12.26 |
| T | G | G | 19 (7.6) | 15 (5.0) | 2.34 | 0.1257 | 0.57 | 0.28-1.17 |
| C | G | A | 20 (8.0) | 1 (0.3) | 22.25 | 0.0000*** | 0.04 | 0.00-0.27 |
| T | A | G | 10 (4.0) | 2 (0.7) | 7.953 | 0.0048** | 0.14 | 0.03-0.68 |
| C | A | G | 42 (16.8) | 1 (0.3) | 46.16 | 0.0000*** | 0.02 | 0.00-0.13 |
| C | A | A | 17 (6.8) | 1 (0.3) | 18.72 | 0.0000*** | 0.04 | 0.00-0.32 |

***p<0.05, **p<0.01, ***p<0.001, NS Non-significant**; CI: confidence interval;

^a^ Fisher’s exact (2x2 table at I df)
